# The effect of perioperative blood transfusion on survival after renal cell carcinoma nephrectomy: A systematic review and meta-analysis

**DOI:** 10.3389/fonc.2023.1092734

**Published:** 2023-02-16

**Authors:** Yang Liu, Xianzhong Deng, Zhi Wen, Jing Huang, Chongjian Wang, Caixia Chen, Xuesong Yang

**Affiliations:** ^1^ Department of Urology, Affiliated Hospital of North Sichuan Medical College, Nanchong, China; ^2^ Department of Urology, Chengdu Xinhua Hospital Affiliated to North Sichuan Medical College, Chengdu, China

**Keywords:** blood transfusion, renal cell carcinoma, survival, systematic review, meta-analysis

## Abstract

**Background:**

The effect of perioperative blood transfusion (PBT) on postoperative survival in RCC patients who underwent partial nephrectomy (PN) or radical nephrectomy (RN) remains controversial. Two meta-analyses in 2018 and 2019 reported the postoperative mortality of PBT patients with RCC, but they did not investigate the effect on the survival of patients. We performed a systematic review and meta-analysis of relevant literature to demonstrate whether PBT affected postoperative survival in RCC patients who received nephrectomy.

**Methods:**

Pubmed, Web of Science, Cochrane, and Embase databases were searched. Studies comparing RCC patients with or without PBT following either RN or PN were included in this analysis. Newcastle-Ottawa Scale (NOS) was used to evaluate the quality of the included literature, and hazard ratios (HRs) of overall survival (OS), recurrence-free survival (RFS), and cancer-specific survival (CSS), as well as 95% confidence intervals, were considered as effect sizes. All data were processed using Stata 15.1.

**Results:**

Ten retrospective studies involving 19,240 patients were included in this analysis, with the publication dates ranging from 2014 to 2022. Evidence revealed that PBT was significantly associated with the decline of OS (HR, 2.62; 95%CI: 1,98-3.46), RFS (HR, 2.55; 95%CI: 1.74-3.75), and CSS (HR, 3.15; 95%CI: 2.3-4.31) values. There was high heterogeneity among the study results due to the retrospective nature and the low quality of the included studies. Subgroup analysis findings suggested that the heterogeneity of this study might be caused by different tumor stages in the included articles. Evidence implied that PBT had no significant influence on RFS and CSS with or without robotic assistance, but it was still linked to worse OS (combined HR; 2.54 95% CI: 1.18, 5.47). Furthermore, the subgroup analysis with intraoperative blood loss lower than 800 ML revealed that PBT had no substantial impact on OS and CSS of postoperative RCC patients, whereas it was correlated with poor RFS (1.42, 95% CI: 1.02-1.97).

**Conclusions:**

RCC patients undergoing PBT after nephrectomy had poorer survival.

**Systematic Review Registration:**

https://www.crd.york.ac.uk/PROSPERO/, identifier CRD42022363106.

## Introduction

Renal cell carcinoma (RCC) accounts for 2%-3% of adults with malignant tumors, second only to prostate cancer and bladder cancer while this disease has the highest mortality in the urinary system tumors (1). The incidence of RCC is surprisingly higher in developed countries, at approximately 3.8% in adults (2). A typical treatment strategy for RCC is the surgical resection of the primary tumor (3). Since RCC is characterized by significant angiogenic activity compared to other diseases, this procedure may result in a large volume of blood loss (4). Under the circumstances, renal cancer patients undergoing nephrectomy are prone to require allogeneic perioperative blood transfusion (PBT). The association has been reported between PBT and tumor recurrence, including colorectal, gastric, pancreatic, liver, prostate, and bladder cancers (5–13). Despite the fact that the underlying mechanism of this association remains unclear, researchers have hypothesized that blood transfusion may be responsible for the immunomodulatory effects and inflammatory responses (14). It is still controversial whether PBT results in poorer survival rates for RCC patients after nephrectomy (15–21). This controversy is probably due to research limitations such as a relatively small sample size, a short period of follow-up (20), insufficient data on potential confounders, the inclusion of heterogeneous RCC histological subtypes, and a lack of information on the timing or volume of blood transfusions (15, 21). Based on the literature published as of October 2022, two NRCT studies have evaluated the prognostic role of PBT in patients undergoing radical nephrectomy (RN) through systematic review and meta-analysis. Both have reported increased mortality in patients receiving PBT. However, such systematic reviews focused only on mortality as an outcome indicator instead of the survival rates of the patients.

The present work is the first systematic review and meta-analysis to comprehensively assess the effect of PBT on postoperative survival in RCC cases. It aims to assess clinicians’ decisions regarding the PBT management of RCC patients by evaluating relevant literature on this research topic, hoping to make some improvements in certain research directions.

## Methods

We conducted a systematic review and meta-analysis following the guidelines of the Preferred Reporting Items for Systematic Reviews and Meta-Analyses (PRISMA). A relevant protocol has been registered on the Prospero website (https://www.crd.york.ac.uk/PROSPERO/) with the registration number: CRD42022363106.

### Literature search, and inclusion, and exclusion criterion

Pubmed, Cochrane Library, Web of Science, and Embase databases were searched as of October 10, 2022. The literature search adopted subject headings combined with free words. The subject terms included renal cancer, renal cell carcinoma, perioperative blood transfusion, radical nephrectomy, and partial nephrectomy. The literature to be searched was limited to clinical trials and those written in the English language. Meanwhile, meta-analyses, conference abstracts, animal experiments, publications written in other languages other than English, and pathological reports were excluded. The participants were diagnosed with RCC and underwent nephrectomy, and the intervention was allogeneic PBT. Patients who received autologous blood or no transfusion and those who developed or had a history of metastatic disease or tumors other than kidney cancer were ineligible. References of the included studies and previous meta-analyses were also reviewed to identify possible eligible studies. Literature screening and retrieval were performed independently by two researchers, and dissents were resolved through discussion.

### Outcome measures and data collection

The primary outcome was overall survival (OS), and the secondary outcomes included RFS and CSS. OS was defined as the duration of time from surgery to death due to any causes. RFS was defined as the duration of time from surgery to the recurrence of cancer. CSS was defined as the duration of time from surgery to death due to cancer recurrence or metastasis. Data were extracted and collected independently by two authors, and disagreements were resolved through discussion to reach a consensus.

### Quality assessment

The quality of the included studies was assessed using the Newcastle-Ottawa Scale (NOS) (https://www.ohri.ca//programs/clinical_epidemiology/oxford.asp). The evaluation details are presented in the **Supplementary Material**; and the quality of the data extracted from each study was assessed based on case selection, comparability, and outcome reporting. Quality assessment was performed independently by two researchers, and disagreements were resolved through discussion.

### Statistical analysis

Meta-analysis was carried out using the Stata 15.1 software (StataSE, USA). The hazard ratios (HRs) with 95% confidence intervals (CIs) for OS, RFS, and CSS were calculated. When both univariate and multivariate analyses were available, the HRs were extracted from multivariate analyses. Engauge Digitizer 4.1 and Adobe Photoshop software were applied for the HR extraction (22). Statistical heterogeneity was evaluated using *I* ^2^ statistics; the value of *I* ^2^ ≥ 50% (*P* ≤ 0.1) indicated a high level of heterogeneity, and a random effects model was employed; when the value of *I* ^2^ < 50%, a fixed effects model was used (*P* > 0.1). A *P* < 0.05 was considered statistically significant (23). Subgroup and sensitivity analyses were performed to explore the source and degree of heterogeneity among studies, if necessary. Egger’s test and funnel plots were adopted to determine publication bias, and a *P* ≥ 0.05 indicated that publication bias had no statistical significance.

## Results

### Literature screening and the characteristics of the included studies

We initially screened a total of 1,295 potential articles, of which 565 were from PubMed, 37 from Cochrane Library, 25 from Embase, 668 from Web of Science, and 3 from other sources.

**Figure 1:**
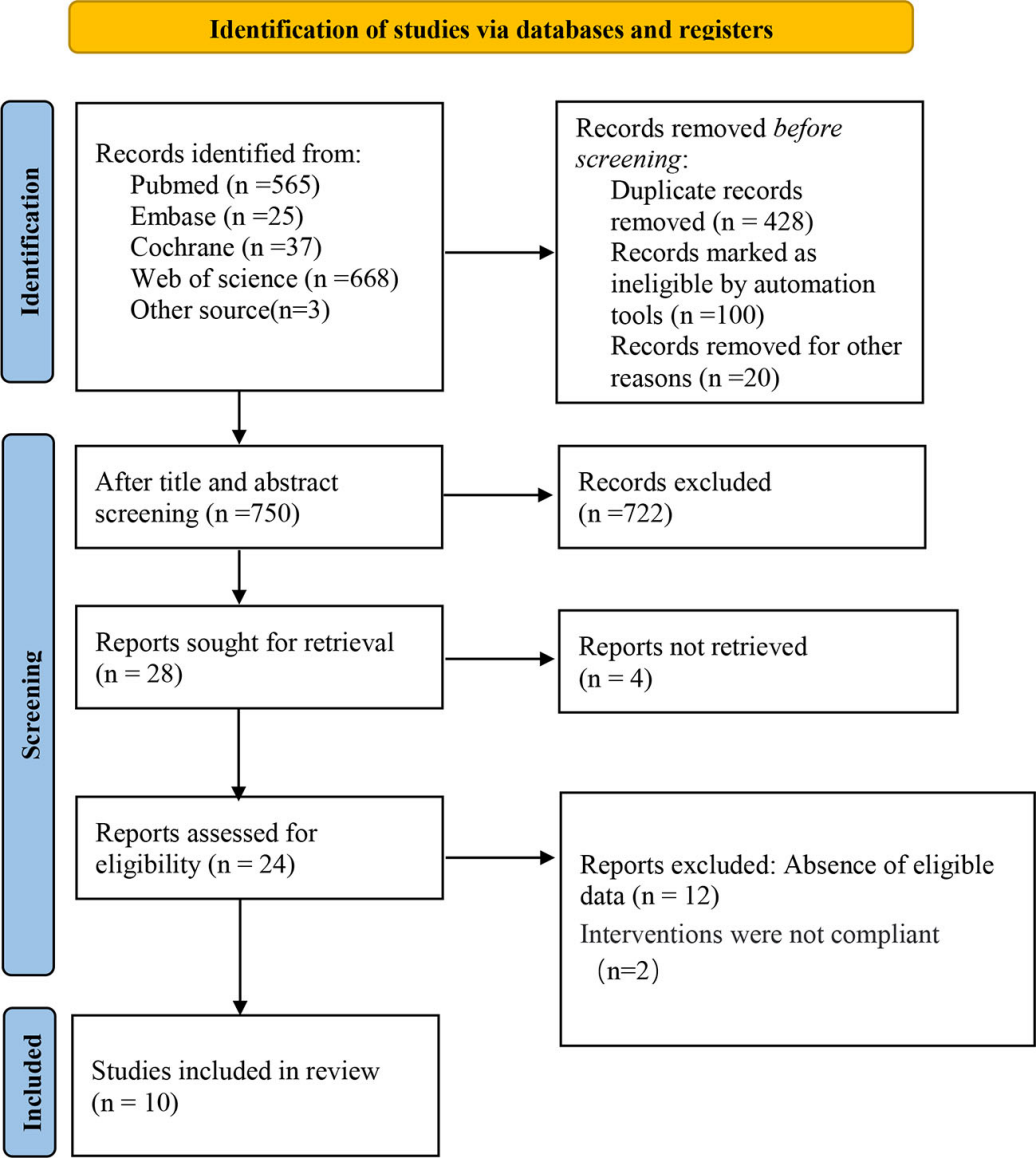
Literature screening flowchart.

A total of 28 relevant articles were obtained after the initial screening. Based on further screening, ten clinical observational studies involving 19,240 patients were ultimately included. The specific literature screening process is shown in **Figure 1**. The ten included studies were conducted in different countries and published between 2014 and 2022. Two were from the United States, four were from South Korea, two were from Israel, one was from Austria, and the last was from France. The characteristics of the included studies are shown in **Table 1**. The median NOS for reporting OS was 7 in the studies, that of reporting RFS was 6, and that of CSS was 7.

**Table 1:** Table of basic information extracted from the literature.

Author (year)	Country	Research time	Article type	sample size	Surgical pathway	Blood transfusion volume (median)	Definition of perioperative period	PT1-T2 to total sample size	Preoperative distant metastasis	Blood loss (pbt\no pbt)	Outcome	NOS
(17)	United States	1990-2006	retrospective study	2318	patency+ telescope	3	Intraoperative; postoperative	79%	exclusion	878\200	OS,CSS	7
(24)	United States	2000-2010	retrospective study	1056	patency+ micro-invasive	2	Intraoperative; postoperative	84%	exclusion	1000\200	OS,RFS,CSS	7
(25)	South Korea	2000-2014	retrospective study	2329	patency+ micro-invasive	3	preoperative,intraoperative and postoperative period	NA	NA	NA	OS,RFS,CSS	5
(26)	South Korea	2000-2014	retrospective study	2329	patency+ micro-invasive+robot	3	preoperative,intraoperative and postoperative period	88.60%	exclusion	700\200	OS,CSS	5
(20)	France	2000-2016	retrospective study	382	patency+ micro-invasive+robot	2	preoperative,intraoperative and postoperative period	74%	exclusion	900\250	OS,RFS,CSS	6
(18)	Israel	1988-2013	retrospective study	1159	patency+ micro-invasive	2	Intraoperative; postoperative	77.50%	exclusion	NA	OS,RFS,CSS	7
(27)	South Korea	1988-2015	retrospective study	4019	patency+ micro-invasive	NA	Intraoperative; postoperative	90%	exclusion	662.9\168	RFS,CSS	6
(28)	Israel	1988-2013	retrospective study	1168	patency+ micro-invasive	2	Intraoperative; postoperative	76.90%	exclusion	NA	RFS,CSS	7
(15)	South Korea	NA	retrospective study	3832	patency+ micro-invasive+robot	NA	preoperative,intraoperative and postoperative period	83.90%	19.9\18.9	800\300	OS	7
(19)	Austria	2004-2014	retrospective study	648	patency+ micro-invasive	2	Intraoperative; postoperative	57%	16.1\12.6	NA	OS,CSS	7

NA, Not Applicable.

### Results of meta-analysis

#### Overall survival

Eight studies reported OS. Data on the occurrence time of events in all eight studies were available. Seven studies were adjusted for multivariate analysis. The adjusted data from three studies were directly extracted from the texts. However, the missing HR data of the remaining five studies were extracted using Engauge Digitizer 4.1 and Adobe Photoshop. One (20) study was excluded from multivariate analysis, and OS data were extracted only from univariate analysis.

**Figure 2:**
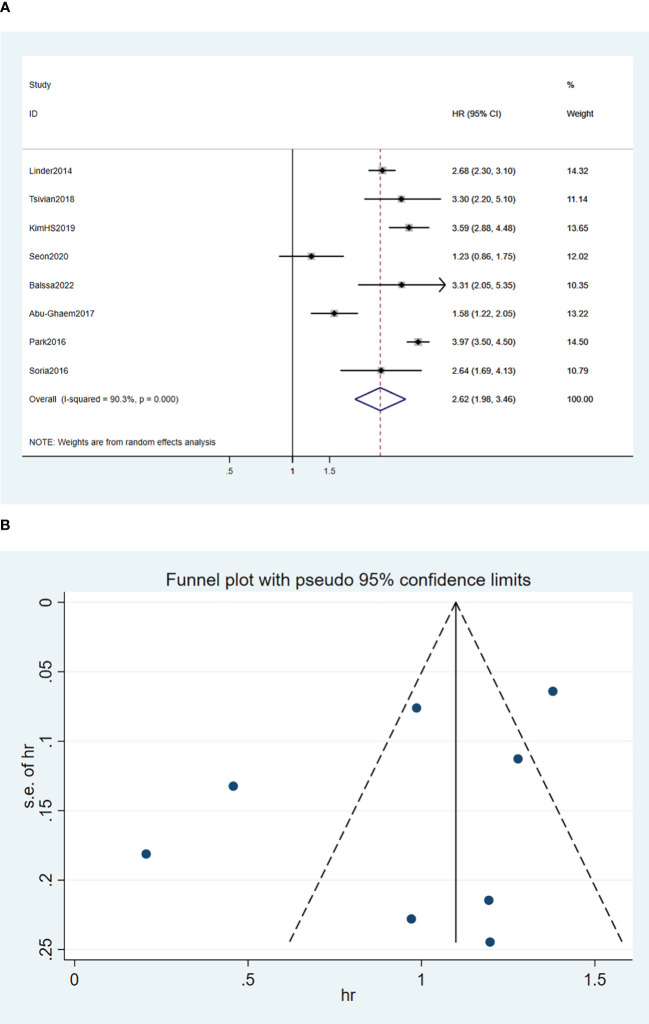
Forest plot **(A)** and funnel plot **(B)** depicting the association between PBT and overall survival. HR values greater than 1 indicate that the intervention is detrimental to survival, implying that PBT was associated with poorer OS.

A random-effects model was used for the meta-analysis of the eight articles (15, 17–20, 24–26), and the results implied that blood transfusion was significantly associated with decreased OS (HR, 2.62; 95% CI: 1,98-3.46; *I* ^2^ = 90.3%, *P* < 0.0001; **Figure 2A**).

#### Recurrence-free survival

Seven studies reported RFS. The data on the occurrence time of events were available in all seven studies, six of which were adjusted for multivariate analysis. The adjusted data of four studies were directly extracted from the texts, and the missing HR data of the rest three studies were extracted using Engauge Digitizer 4.1 and Adobe Photoshop. One study (20) was excluded from multivariate analysis and a single variable was adopted to analyze the data.

**Figure 3:**
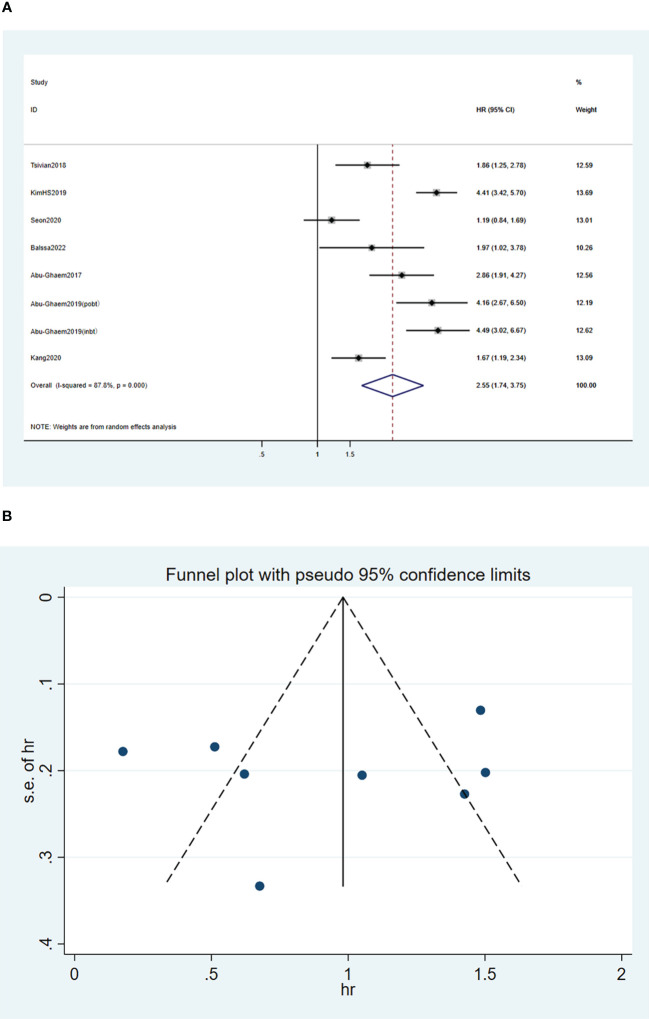
Forest plot **(A)** and funnel plot **(B)** depicting the association between PBT and recurrence-free survival. HR values greater than 1 indicate that the intervention is detrimental to survival, implying that PBT was associated with poorer RFS.

A random-effects model was used for the meta-analysis of the seven articles (18, 20, 24–28), and the results indicated that blood transfusion was significantly associated with reduced RFS (HR, 2.55; 95%CI: 1.74-3.75; *I^2^* = 87.8%, *P* < 0.001; **Figure 3A**).

#### Cancer-specific survival

Nine studies reported CSS. The occurrence time of events was available in the nine studies, and the studies were adjusted for multivariate analysis. The adjusted data of three studies were directly extracted from the texts while the missing HR data of the rest six studies were extracted using Engauge Digitizer 4.1 and Adobe Photoshop software.

**Figure 4:**
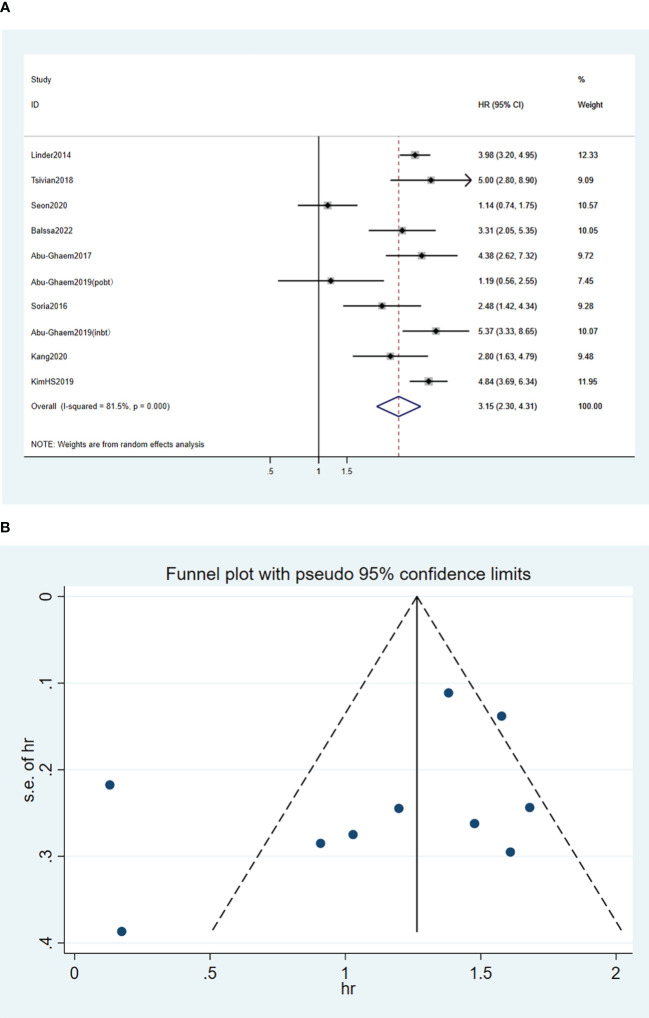
Forest plot **(A)** and funnel plot **(B)** depicting the association between PBT and cancer-specific survival. HR values greater than 1 indicate that the intervention is detrimental to survival, implying that PBT was associated with poorer CSS.

A random effects model was used for the meta-analysis of the nine articles (17–20, 24–28), and the results indicated that blood transfusion was positively associated with decreased CSS (HR, 3.15; 95%CI: 2.3-4.31; *I^2^* = 81.5%; *P* < 0.001; **Figure 4A**).

#### Subgroup analysis

**Figure 5:**
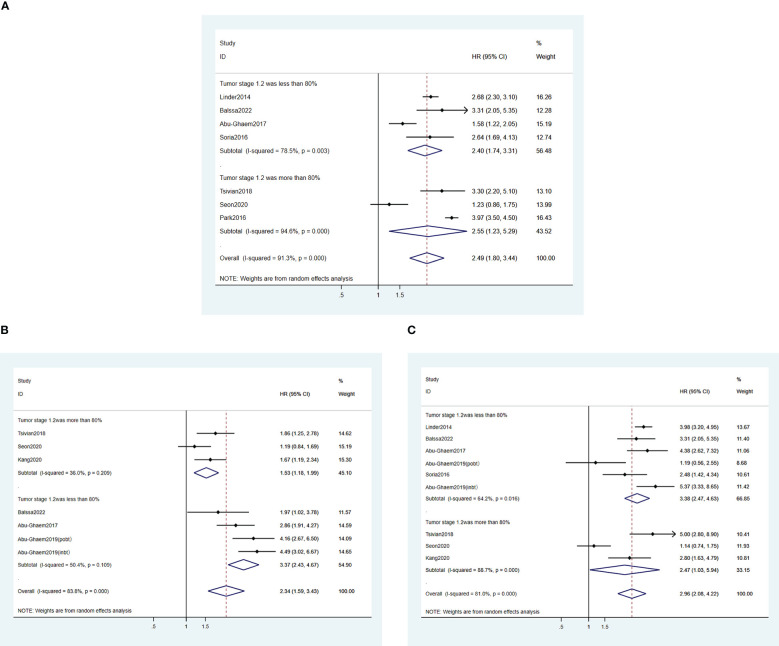
Forest plot depicting the association between **(A)** tumor staging and OS; **(B)** tumor staging and RFS; **(C)** tumor staging and CSS. An HR value greater than 1 is detrimental to survival, implying that tumor staging did not affect PBT and poorer postoperative survival.

To determine the heterogeneity of this meta-analysis, subgroup analyses were performed according to the characteristics among the included studies (e. g. early T1-T2 tumor sample proportion, surgical pathway, blood transfusion volume, year of publication, blood loss volume, definitions of perioperative period, and the occurrence of distant metastasis of the enrolled patients). **Tables 2**–**4** show the subgroup analyses of CSS, OS, and RFS, respectively. The subgroup analysis of RFS was conducted according to whether the patients at tumor stages 1-2 exceeded 80% of the total sample size. The results indicated that no RFS (HR, 1.53 95%CI: 1.18-1.99; *I^2^* = 36%) was reported in the over 80% group, which was better than the below 80% group (HR, 3.37 95%CI: 2.43-4.67), *I^2^* = 50.4%), and the heterogeneity of results was greatly reduced (**Figure 5B**). In contrast, the heterogeneity of the results of OS and CSS did not reduce (**Figures 5A, C** **)**. A robot-assisted surgical approach was reported in only three studies. Subgroup analysis revealed that the application of robot-assisted surgery was not a source of heterogeneity in the outcomes of the studies. Additionally, two studies reported intraoperative blood loss lower than 800 ML. Subgroup analysis of blood loss volume showed that the differences in blood loss volume between studies were also not the source of heterogeneity. One study reported RFS at different tumor stages (24), and only one study presented the postoperative survival of patients who underwent different surgical options (partial vs. total nephrectomy) (27). It is therefore not sufficient to perform a subgroup analysis of tumor stage and surgery. The remaining subgroup analyses also did not reduce the outcome heterogeneity. These subgroup analyses revealed an association between PBT and poor OS, RFS, and OS, implying that PBT was correlated with poor survival outcomes in RCC patients with the described characteristics.

**Table 2:** Subgroup analysis of cancer-specific survival.

Stratified	No. of studies	I^2^	HR (95%CI)	P value
Publication date				
2014-2016	2	58.20%	3.38 (2.17-5.25)	P=0.122
2016-2022	8	84.70%	3.1 (2.02-4.75)	P<0.001
Blood loss volume				
<800	2	84.80%	1.76 (0.73-4.23)	P=0.01
≥800	3	0.00%	3.96 (3.29-4.78)	P=0.56
Neoplasm staging				
Tumor stage 1.2 was less than 80%	6	64.20%	3.38 (2.47-4.63)	P=0.016
Tumor stage 1.2 was more than 80%	3	88.70%	2.47 (1.03-5.94)	P<0.001
Distant metastasis				
No distant metastasis	8	83%	3.02 (2.05-4.46)	P<0.001
Distant metastasis	1	NR	2.48 (1.42-4.34)	NR
Perioperative definition				
Intraoperative and post opertive	7	62.10%	3.47 (2.59-4.67)	P=0.015
Preoperative, intraoperative and post operative	3	93.70%	2.65 (1.1-6.43)	P<0.001
Blood transfusion volume				
The volume of blood transfusion was 3 units	3	94%	2.87 (1.44-5.71)	P<0.001
The volume of blood transfusion was 2 units	6	65.60%	3.4 (2.32-4.98)	P=0.013
Surgical pathway				
Open and minimally	8	62%	3.71 (2.89-4.76)	P=0.01
Open minimally and robotic	2	90.60%	1.93 (0.68-5.5)	P=0.001

**Table 3:** Subgroup analysis of overall survival.

Stratified	No. of studies	I^2^	HR (95%CI)	P value
Publication date				
2014-2016	3	88%	3.11 (2.26-4.28	<0.0001
2016-2022	5	90.10%	2.36 (1.49-3.74	<0.0001
Blood loss volume				
<800	1	NR	1.23 (0.86-1.75)	NR
≥800	4	80.80%	3.28 (2.53-4.26)	p=0.001
Neoplasm staging				
Tumor stage 1.2 was less than 80%	4	78.50%	2.4 (1.74-3.31)	p=0.003
Tumor stage 1.2 was more than 80%	3	94.60%	2.55 (1.23-5.29)	p<0.0001
Distant metastasis				
No distant metastasis	5	86.20%	2.21 (1.54-3.16)	p<0.001
Distant metastasis	2	66.30%	3.43 (2.34-5.03)	p=0.085
Perioperative definition				
Intraoperative and postopertive	4	79.20%	2.42 (1.76-3.32)	p=0.002
Preoperative, intraoperative and postopertive	4	92%	2.8 (1.79-4.38)	p<0.001
Blood transfusion volume				
The volume of blood transfusion was 3 units	3	92.10%	2.34 (1.46-3.73)	p<0.001
The volume of blood transfusion was 2 units	4	77.40%	2.53 (1.68-3.82)	p=0.004
Surgical pathway				
Open and minimally	5	83%	2.64 (1.98-3.52)	p<0.001
Open minimally and robotic	3	94.60%	2.54 (1.18-5.47)	p<0.001

**Table 4:** Subgroup analysis of recurrence-free survival.

Stratified	No.of studies	I^2^	HR (95%CI)	P value
Blood loss volume				
<800	2	45.50%	1.42 (1.02-1.97)	0.175
≥800	2	0	1.89 (1.34-2.66)	0.886
Neoplasm staging				
Tumor stage 1.2 was less than 80%	4	78.50%	2.4 (1.74-3.31)	P=0.003
Tumor stage 1.2 was more than 80%	3	94.60%	2.55 (1.23-5.29)	P<0.001
Perioperative definition				
Intraoperative and postopertive	5	81%	2.75 (1.84-4.13)	P<0.001
Preoperative, intraoperative and postopertive	3	94.50%	2.2 (0.86-5.61)	P<0.001
Blood transfusion volume				
The volume of blood transfusion was 3 units	2	97.20%	2.31 (0.64-8.31)	P<0.001
The volume of blood transfusion was 2 units	5	69.70%	2.93 (2.04-4.21)	P=0.01
Surgical pathway				
Open and minimally	6	84.20%	3 (2.07-4.36)	P<0.001
Open minimally and robotic	2	43%	1.42 (0.89-2.25)	P=0.186

#### Publication bias and sensitivity analysis

Funnel plots of the three outcome-Funnel plots of the three outcome (**Figures 2B**, **3B**, **4B**) indicators were asymmetry to the naked eye. Egger’s tests indicated no significant publication bias in OS (*P* = 0.248), RFS (*P* = 0.569), and CSS (*P* = 0.239). A *P* > 0.05 indicated that the publication bias was not significant. Sensitivity analyses revealed that the removal of any one article produced no significant influence on the results.

## Discussion

To our knowledge, the current study is the first meta-analysis to systematically evaluate the influence of PBT on survival outcomes in patients undergoing RCC surgery. Ten eligible studies were identified, involving more than 19,000 patients. The meta-analysis revealed that PBT was correlated with decreased OS (HR, 2.62; *P* < 0.01), RFS (HR, 2.55; *P* < 0.001), and CSS (HR, 3.15; *P* < 0.001) in RCC patients undergoing nephrectomy (i.e., non-metastatic and metastatic RCC).

Tumor recurrence and metastasis are associated with inflammation and immune regulation (29, 30). The allogeneic PBT may accelerate tumor progression *via* immune responses, the reaction of cytokine degradation of lipid membrane release, and other non-immunogenic mechanisms (31). Keown et al. have called the immune regulation mechanism caused by blood transfusion as transfusion-related immune regulation (TRIM). These immune regulation mechanisms include immunosuppression and proinflammatory effects mediated by residual leukocytes, apoptotic cells, and modified biological responses (e.g. cytokines, soluble mediators, and soluble HLA peptides) (31). It may produce adverse consequences such as CMV or HIV reactivation, red blood cell alloimmunization, and increased cancer recurrence (21, 32). This suggested that blood transfusion was independently associated with an increased risk of disease recurrence and compromised postoperative survival.

PBT has been reported to be associated with poor postoperative survival outcomes in some cancers (8, 33–36). It also produces a significant negative impact on long-term prognosis and increased short-term complications after colorectal cancer surgery (35). Annamaria Agnes et al. have uncovered a negative association between PBT and OS, DFS, and DSS in gastric cancer, and Moschini et al. have found that PBT was significantly correlated with adverse postoperative survival in urinary tumor patients undergoing radical bladder cancer surgery, and so does radical prostatectomy according to Kim et al. (8, 33, 34). Besides, ABU et al. have also revealed adverse effects of PBT on survival after nephrectomy (18). The results of this meta-analysis are consistent with the findings of previous literature, indicating the presence of an association between PBT and OS, RFS, and CSS. The final meta-analysis demonstrated a negative correlation between PBT and the survival indicators in RCC patients following nephrectomy.

Two previous meta-analyses (37, 38) have focused only on mortality as the outcome indicator, but our research is the first meta-analysis on survival, which fills the gap in this field and provides some references for clinical decision-making. However, there are still some limitations in the present research. First, despite a comprehensive search, the number of the included studies and samples was rather limited. Some of the included studies were from the same country and even the same unit, which might cause an overestimation of the effect of blood transfusion on survival rates. Meanwhile, all the included studies were designed retrospectively, and there were biases in reporting, selection, confirmation, and measurement. All this might cause high heterogeneity in our results. Being aware of the heterogeneity of the included literature, we conducted subgroup analyses to identify the source of heterogeneity among studies according to the proportion of early T1-T2 tumors, surgical pathway, blood loss volume, publication year, blood transfusion volume, definitions of perioperative periods, and the occurrence of distant metastasis in the included samples. However, due to limited data, subgroup analyses on tumor staging and surgical pathways could not be carried out. It is of great clinical significance, and thus more attention shall be paid to these data updated in the future. According to the subgroup analysis of the RFS, the proportion of early tumors might be the source of heterogeneity in the included studies.

Due to the heterogeneity caused by statistical methods among studies, confounding factors could not be excluded. Furthermore, various surgical techniques and patient case combinations might cause additional biases. Experiences of surgeons might be a confounding factor, and PBT decisions in the included literature were mostly made as per the experience of clinicians (surgeons and/or anesthesiologists), without a recognized transfusion criterion. Finally, the included articles had both single-center and multi-center data, whereas not all reported survival indicators. Engauge Digitizer 4.1 and Adobe Photoshop software were applied for HR extraction, which may have potential errors. Although the random-effects model took into account heterogeneity among studies, the conclusions should be interpreted with caution. In the current meta-analysis, funnel plots revealed the presence of reporting bias or an overestimation of the effect of PBT on survival outcomes.

In summary, this study confirmed that PBT had a significant negative correlation with survival rates in patients undergoing RCC nephrectomy, and further research is required to support and verify the findings.

## Conclusions

PBT might be associated with reduced survival rates in RCC patients undergoing nephrectomy. Given the low quality of the extracted data and a high level of heterogeneity among the included studies, more prospective high-quality studies are needed to provide specific guidelines on PBT.

## Data availability statement

The original contributions presented in the study are included in the article/**Supplementary Material**. Further inquiries can be directed to the corresponding author.

## Author contributions

YL, XD, and XY contributed to conception and design of the study. ZW and JH organized the database. YL and XD performed the statistical analysis. YL, XD, and XY wrote the first draft of the manuscript. ZW, JH, CW, and CC wrote sections of the manuscript. All authors contributed to manuscript revision, read, and approved the submitted version.
