# Supplementary material for: The effect of perioperative blood transfusion on survival after renal cell carcinoma nephrectomy: A systematic review and meta-analysis
Source: Front Oncol. 2023 Feb 16;13:1092734. doi: 10.3389/fonc.2023.1092734 (PMC9978807; doi:10.3389/fonc.2023.1092734)
Supplement: Supplementary file 1 [file Table_1.docx]

**Table S1** **|** Quality evaluation.

| NOS | | | | Selection | | | | Comparability | Outcome | | | Overall score |
| --- | --- | --- | --- | --- | --- | --- | --- | --- | --- | --- | --- | --- |
| ID | First Author | Year | Study design | Representativeness of the exposed cohort | Selection of the nonexposed cohort | Assessment of exposure | Demonstration that outcome of interest was not present at start of study | Comparability of cohorts on the basis of the design or analysis | Ascertainment of outcome | Long enough follow-up for outcomes to occur | Adequacy of follow-up of cohorts |  |
| Linder2014 | brianJ.Linder | 2014 | cohort study | A | B | A | A | A | A | A | A | 7 |
| M.D2018 | matvey Tsivian,M.D | 2018 | cohort study | A | B | B | A | A | A | A | A | 7 |
| Abu-ghanem2019 | Yasmin Abu-Ghanem | 2019 | cohort study | A | B | A | A | A | A | A | A | 7 |
| kim,H.s2019 | Kim,H.S | 2019 | cohort study | A | C | C | A | A | A | A | A | 5 |
| kang2020 | HO Won kang | 2020 | cohort study | A | B | B | A | A | A | A | A | 6 |
| seon2020 | DONG Young Seon | 2020 | cohort study | A | C | B | A | A | B | A | A | 5 |
| L.BALSSA2022 | P. Détréea,b, L. Balssa | 2022 | cohort study | A | C | C | A | A | A | A | A | 6 |
| Abu-ghanem2017 | Yasmin Abu-Ghanem | 2017 | cohort study | A | B | A | A | A | A | A | A | 7 |
| PARK2016 | Yong Hyun Park | 2016 | cohort study | A | B | A | A | A | A | A | A | 7 |
| Soria2016 | Francesco Soria | 2016 | cohort study | A | B | A | A | A | A | A | A | 7 |
